# Duramesh™ versus conventional suture for prevention of trocar-site hernia following laparoscopic surgery (TROCAR): study protocol for a double-blind randomised controlled trial

**DOI:** 10.1186/s13063-025-09388-3

**Published:** 2025-12-30

**Authors:** Lawrence Nip, Sarah Zhao, Rhys Thomas, Paul Bassett, David Ross, Alastair C. J. Windsor, Chris Brew-Graves, Steve Halligan, Samuel G Parker

**Affiliations:** 1 The Abdominal Wall Unit, Croydon University Hospital, 530 London Road, Thornton Heath, London, CR7 7YE UK; 2 Division of Medicine, Centre for Medical Imaging, The Rayne Institute, University College London, 5 University Street, London, WC1E 6JF UK; 3 Statsconsultancy Ltd, 40 Longwood Lane, Amersham, Buckinghamshire, HP7 9EN UK; 4 The London Clinic, 20 Devonshire Place, London, W1G 6BW UK; 5 The Princess Grace Hospital, 42-52 Nottingham Place, London, W1U 5NY UK; 6 National Cancer Imaging Translational Accelerator (NCITA), The Rayne Institute, University College London, 5 University Street, London, WC1E 6JF UK

**Keywords:** Duramesh, Mesh suture, Trocar-site hernia, Hernia prevention, Laparoscopic surgery

## Abstract

**Background:**

Trocar-site hernia is an underappreciated condition with estimates of approximately 25% at 2 years follow-up. Duramesh™ has emerged as a novel product with potential benefits for incisional hernia prevention. The aim of this trial is to establish if Duramesh is superior to conventional suture for prevention of periumbilical trocar-site hernia following laparoscopic surgery.

**Methods:**

The TROCAR trial is a prospective single-centre, parallel arm, double-blind randomised controlled trial conducted in the United Kingdom. A total of 250 randomised participants (1:1 ratio) will be allocated to receive either Duramesh or conventional suture (J-vicryl or J-PDS). The primary outcome is the cumulative incidence of sonographically detected periumbilical trocar-site hernia at 2 years of the index operation. Secondary outcomes are 90-day surgical site occurrence (SSO), 90-day surgical site infection (SSI), 90-day rate of reoperation, 90-day mortality, length of hospital stay, and quality of life at 3 months, 1 year and 2 years measured using a modified EuraHS-QoL score and modified Carolinas Comfort Scale.

**Conclusion:**

TROCAR will provide level 1 evidence on trocar-site hernia prevention in both the emergency and elective settings.

**Trial registration:**

Registration number ISRCTN14473961 (https://doi.org/10.1186/ISRCTN14473961). Registered on 14th April 2025.

**Supplementary Information:**

The online version contains supplementary material available at 10.1186/s13063-025-09388-3.

## Background

During laparoscopic surgery, ports or trocars are inserted into the abdominopelvic cavity to facilitate gas insufflation and access for surgical instruments. Entry into the abdominopelvic cavity typically commences around the umbilicus as postoperative scars are relatively well hidden and the cicatrix can be lifted to reduce the risk of enterotomy. A small incision at the base of the cicatrix and through the adjoining linea alba is required. However, this predisposes to trocar-site incisional hernia (or trocar-site hernia (TSH)) where intraabdominal viscera or fat protrudes through the surgical defect after closure [1]. TSH occurrence is higher when incisions are larger, such as those 10 mm and greater, or extended as a specimen extraction site [2]. TSH are therefore felt to occur more commonly at the periumbilical region as this is conventionally where larger 10 to 12 mm trocars are placed for laparoscopic camera access [1].

There is considerable variation in the reported incidence of TSH from 0 to 39% [3]. A survey of American surgeons found that the perceived rate of TSH for trocar sites 10 mm and above was 5% [3]. However, this is likely an underestimate as much of the available literature consists of retrospective studies with poorly defined follow-up, inadequate follow-up duration and insensitive hernia assessment (for example, using palpation or self-reporting rather than imaging confirmation) [4]. Imaging is known to increase the diagnostic yield as clinically occult herniae are detected [5, 6]. High-quality prospective studies, including those with longer follow-up duration and imaging confirmation, have found the incidence to exceed 25% [7–9].

Periumbilical trocar site closure is usually performed with slowly absorbable suture such as vicryl or polydioxanone (PDS). Most surgeons do not perform a concurrent repair with prophylactic planar mesh as implantation prolongs surgery and developing a retromuscular plane (considered the gold standard for mesh placement) without extending the fascial incision is technically challenging. Some authors investigated prophylactic mesh implantation during laparoscopic surgery but utilised onlay repair [4], which was ineffective for preventing TSH compared to suture closure alone. Alternative options have been explored such as intraperitoneal mesh, but there are concerns this may precipitate adhesions to viscera and enterocutaneous fistulae [10]. A simple solution to prevent TSH is needed, especially since minimally invasive surgery is now the standard of care for many operations.

### Duramesh™

Duramesh is a novel product that combines properties of conventional suture with planar mesh. It gained its Conformité Européenne (CE) mark in May 2021 and United States Food and Drug Administration (FDA) approval in September 2022 and thereafter has been clinically indicated for cases of abdominal wall closure and ventral hernia repair. Specifically, in contrast to conventional suture, which has a single filament, Duramesh comprises multiple filaments that form a permeable cylindrical shape in cross-section. It is implanted and tied in a similar manner to conventional suture, but flattens when tension is applied, at which point it resembles a ribbon of planar mesh.

The mechanism of action of Duramesh is thought to be due to the distribution of tension at the suture tissue interface [11]. This limits the effect of ‘cheesewiring’ which can create defects in tissue that enlarge over time with repeated abdominal wall straining. Duramesh has shown greater resistance to tissue pull-through compared to conventional suture in pre-clinical models [12], and a recent systematic review [13] suggests it may be associated with low rates of incisional hernia at 12 months (median 3.4%, *n* = 553). We aim to perform a prospective comparative study investigating the effectiveness of Duramesh for closing laparoscopic periumbilical trocar sites.

## Methods

### Study design

TROCAR is a single-centre, parallel arm, double-blind randomised controlled trial. The study objective is to assess whether Duramesh is superior to conventional slowly absorbable suture (also known as J-vicryl or J-PDS) for closing laparoscopic periumbilical trocar sites 10 mm or greater.

### Setting

The Sponsor and study centre is Croydon University Hospital, a university affiliated district general hospital in London, part of the National Health Service, United Kingdom (UK). Ten consultant surgeons and two associate specialists will be randomising patients and performing closure of the periumbilical trocar site. Their collective practice includes approximately 750 patients, who undergo laparoscopic surgery annually.

### Primary outcome

The primary endpoint is the cumulative occurrence of trocar-site hernia (TSH) over 2 years following the index operation. For this trial, TSH is defined as an ultrasound-detected fascial defect of 10 mm or greater within the vicinity of the periumbilical scar, with or without protrusion of intraabdominal or pre-peritoneal contents, and with or without symptoms.

### Secondary outcomes

These include:

- Incidence of periumbilical surgical site infection (SSI) within 90 days, categorised by the Centers for Disease Control and Prevention (CDC) [14] as superficial, deep and organ-space.
- Incidence of periumbilical surgical site occurrence (SSO) within 90 days, categorised by the Ventral Hernia Working Group (VHWG) [15] as SSI, seroma, haematoma, superficial dehiscence, fascial dehiscence, enterocutaneous fistula, suture granuloma and chronic sinus tract.
- Reoperation within 90 days of the index operation.
- Length of hospital stay.
- Mortality within 90 days of the index operation.
- Measurement of postoperative quality of life (QoL) using modified versions of validated questionnaires – EuraHS-QoL score [16] and Carolinas Comfort Scale (CCS) [17, 18].

### Eligibility criteria

Inclusion criteria are adults 18 or older; able to provide informed consent without barriers that might prevent follow-up; and any laparoscopic procedure requiring periumbilical incision for trocar insertion ≥ 10 mm, elective or emergency, pertaining to the domain of general surgery. Acceptable situations include extraction sites up to a maximum of 3 cm, concurrent umbilical hernia if the defect is small enough to accommodate the camera port, and previous abdominal surgery except for the condition specified in the exclusion criteria below.

Exclusion criteria at screening are lack of capacity to provide informed consent; planned open operation; procedures where placement of periumbilical trocar < 10 mm is planned; periumbilical trocar insertion not planned or not possible; specimen extraction site at the umbilicus exceeds 3 cm; use of planar mesh to cover the periumbilical trocar site; previous planar mesh covering the periumbilical trocar site; previous abdominal surgery, if in the opinion of the Principal Investigator, is not suitable for inclusion, e.g. ‘battlefield’ abdomen, enterocutaneous fistulae, multiple previous laparotomies etc.; CDC wound class 4; pregnancy; and body mass index (BMI) ≥ 40.

For elective and expedited surgery, patients fulfilling inclusion criteria and not meeting exclusion criteria will be offered the opportunity to participate in advance by researchers screening operating lists. Patients undergoing emergency surgery will be offered the opportunity to participate by the consultant surgeon on call when the decision for laparoscopic surgery is declared.

### Standardisation

Training sessions for participating surgeons were conducted to facilitate standardisation of technique when entering and closing the abdomen. An open Hasson cut-down approach and a vertical midline incision in the linea alba are mandated. Fascial stay sutures are permitted but must not contribute to the final fascial closure. Once the operation is complete, closure will be performed with a series of simple interrupted sutures. The number of simple interrupted sutures required will depend on the size of the incision, as some incisions will need to be extended to extract the specimen (up to a maximum of 3 cm). As a result, the midline incision will be closed vertically. Other than the closure method, there is no difference in perioperative management between intervention and control groups.

### Intervention arm

These patients will have interrupted closure with size 1 Duramesh on a 26 mm needle, product code MSP300-5. The number of interrupted sutures along the length of the wound, required to achieve a robust closure, will be at the discretion of the operating surgeon, but bites are recommended to be taken approximately 8 mm from the fascial edge and approximately 8 mm apart. The number of throws required to secure each Duramesh stitch should be exactly 5. After each final throw, the knot should be pulled and pressed down by the operating surgeon, adding a little extra force to the mesh suture ends. The knot may be buried at the surgeon’s discretion in low BMI participants or if there are concerns about a suture granuloma or chronic sinus tract. This is performed by taking a further bite into the subcutaneous fat to invert the knot away from the skin surface. The ears of the knot should be cut to approximately 3 to 4 mm in length, as recommended by the manufacturer. The skin is closed according to surgeon preference. An adhesive dressing or skin glue will be applied over this, again according to surgeon preference.

### Control arm

Participants in the control arm will have interrupted fascial closure with either 0 vicryl or 0 PDS suture, also termed ‘J-vicryl’ or ‘J-PDS’. As for the intervention arm, the number of interrupted sutures required to close the fascial incision will depend on its size and at the discretion of the operating surgeon to achieve a robust closure free of palpable defects. Bites should be placed approximately 5 to 10 mm from the facial edge and 5 to 10 mm apart. The number of throws will be at the discretion of the operating surgeon. Skin closure and skin dressings or glue will be conducted in the same manner as the intervention group.

### Participant timeline (Table 1) and baseline data

**Table 1:**
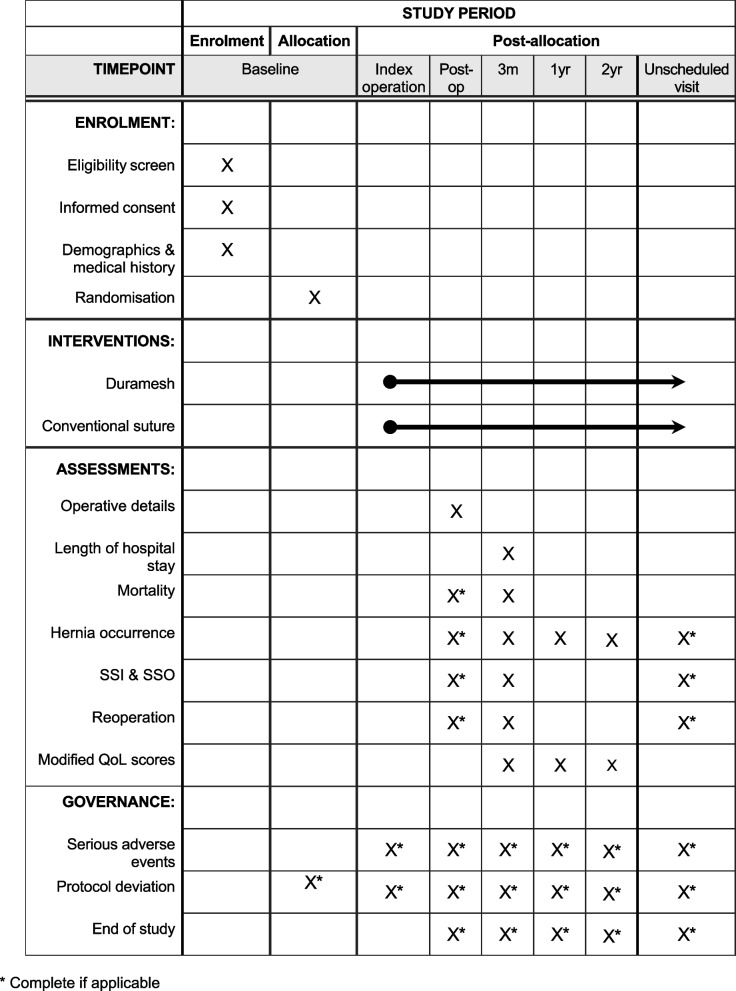
Scheduled and unscheduled visits with timepoints for data collection

Data will be recorded using electronic case report forms (eCRFs) on a secure web-based system (Project REDCap, Vanderbilt University, TN, USA). These consist of baseline demographics, medical history, treatment group, operative details, and primary and secondary outcomes. Follow-up timepoints are 3 months, 1 year and 2 years after the index operation. Additionally, patients requiring inpatient admission will be reviewed by a researcher before discharge for wound events as defined previously.

Baseline demographic data includes age, gender, smoking status, and previous abdominal surgery. Medical history includes history of diabetes, active malignancy, medications affecting healing (chemotherapy, steroids, biologics), ASA grade and comorbidities. Operative details include responsible consultant, pre-operative antibiotics, laparoscopic operation, length of procedure, CDC wound classification, and material used for skin closure.

### Three-month follow-up

Participants are contacted by telephone to arrange a suitable time to visit the outpatient clinic. Information gathered will include length of hospital stay, readmissions within 90 days, return to theatre within 90 days, SSO within 90 days and QoL using modified versions of validated questionnaires. Furthermore, participants will have physical and ultrasound examinations performed by a trained sonographer for the presence of TSH. In this trial, imaging is the definitive modality for detecting the primary outcome. The precise extent (in mm) of any defect, if present, will be recorded, as will whether there is any hernia through this. If so, the width and craniocaudal diameter of the hernia sac will be recorded, along with the presence of any content beyond fat.

### One-year and 2-year follow-up

Data collected at these timepoints are QoL scores and presence of TSH. Ultrasound examination will be performed by the same sonographers who remain blinded to the participant treatment arm. Patients with confirmed TSH detected before the primary endpoint of 2 years are offered ongoing follow-up to monitor TSH size and continued QoL assessment.

### Standard procedure for ultrasound examination

Sonographers consist of one surgical trainee (LN) and two consultant radiologists who will perform focused periumbilical ultrasound. Sonographers will use either a portable probe (Vscan Air, GE Healthcare) connected to an iPad, or a formal machine (LOGIQ E10, GE Healthcare) based on availability. A linear probe will be used except in cases of high BMI where the depth of subcutaneous fat requires a curvilinear probe to visualise the linea alba. Patients will be examined in the supine position with hands placed behind the head, at rest and with Valsalva manoeuvre.

LN will undergo training sessions by the consultant radiologists until deemed competent. Pairs of raters will assess each patient until hernias are detected in 10 participants. Thereafter, scans will be performed independently. Agreement between raters will be assessed using the kappa statistic. A third blinded consultant radiologist will adjudicate for ambiguous cases. Images will be retained for inter-rater reliability testing.

### Sample size

This study will be powered for detecting a difference in the primary outcome between groups. Previous prospective studies with similar follow-up duration record an incidence of trocar-site hernia in the region of 25% [7–9]. An absolute reduction in primary outcome of 15% is expected within the intervention group, down to an occurrence of 10%. The best available evidence comes from a systematic review of Duramesh implantation for laparotomy and ventral hernia repair with a median incisional hernia rate of 3.4% after 1 year [13]. An occurrence of 10% was selected as a conservative estimate of the effect size in the intervention group after 2 years. Therefore, using a significance level of 5% and power of 80%, it is calculated that 100 patients per group, 200 in total, are required. After factoring in a 20% rate of loss to follow-up, a total of 250 participants will be included in the trial.

### Recruitment

Potential participants will be selected from local elective, expedited or emergency pathways. Theatre lists will be screened daily for eligible patients undergoing laparoscopic surgery. Patients will be sent participant information leaflets in advance where possible. Informed consent will be obtained on the day of surgery by a member of the research team. A prospective screening log of patients not eligible or who declined participation will be maintained to monitor selection bias.

### Randomisation

Patients will be randomly allocated to either the intervention or control arm when they arrive in the anaesthetic room before knife to skin. The randomisation sequence was generated and is held by the trial statistician. Randomisation and assigning the intervention are performed by the operating consultant surgeon using a secure web-based system (sealedenvelope.com, Sealed Envelope Ltd., London, UK) ensuring allocation concealment. Following statistical advice, the computer-generated randomisation sequence (in 1:1 ratio) will use a block size of 4 and stratify according to BMI (≥ 30 or < 30) and diabetes (present or absent). The operating consultant surgeon will then close the trocar site with the material specified by the allocation arm.

### Blinding

Both participants and outcome assessors are blinded to the treatment arm during the entire study period. By definition, surgeons performing the operation cannot be blinded. To avoid bias, operating surgeons will not be involved in outcome data collection and will be instructed not to reveal the group allocation to researchers or the patients themselves. In addition, they will be instructed not to specify the closure method in the operative records, instead documenting that the fascia was closed according to the TROCAR trial protocol. All other intraoperative and postoperative details will be recorded as usual. Access to data on REDCap will be limited based on blinded, unblinded and administrator roles.

An emergency unblinding process will be available at all hours of the day. Consultant surgeons are permitted to request an emergency unblinding following direct discussion with the Principal Investigator. Unblinding rates will be logged and checked throughout the trial by the research team and Data Monitoring Committee.

### Protocol deviations

Major protocol deviations are defined as those which become necessary to protect participant safety or failure to report a serious adverse event to the Research Ethics Committee (REC) within 14 days. The decision to withhold Duramesh in an intervention group patient or the placement of Duramesh in a control group patient for safety reasons are classified as major protocol deviations. Minor protocol deviations can be managed internally and include out-of-window visits, accidental unblinding and administrative errors. Accidental unblinding will be managed using a different blinded sonographer for outcome assessment. Non-compliance with the allocated treatment arm due to surgeon error is considered an administrative error. Protocol deviations will be documented, and an intention-to-treat analysis will be performed, with major deviations excluded from the per-protocol set. Major deviations are reported to the REC and minor deviations are reported to the Trial Steering and Data Monitoring and Safety Committees.

In the event of a return to theatre deemed unrelated to the method of closure (e.g. complication from intraabdominal compartment), the patient will be re-closed according to the study arm to which they were initially allocated to. For device-related reoperation, the explant of Duramesh can be undertaken if felt to be clinically indicated, and the foreign material will be sent to the laboratory for microbiological and/or histological analysis. After the explant, the fascia will be closed by a method up to the discretion of the operating surgeon.

### Loss to follow-up

Our sample size calculations have factored in a 20% rate of loss to follow up, which is based on other studies in surgical populations requiring 2-year follow-up with imaging to detect incisional hernia [19, 20]. By consenting to participate, patients are consenting to the intervention, assessments, follow-up and data collection. Study participants are free to withdraw at any time.

Participants may be withdrawn from the trial by the research team if continued participation is no longer in their best interests. Reasons for discontinuing may include the following: intercurrent illness making data collection impossible, persistent non-compliance with protocol requirements hampering and/or biasing data collection, participant fulfils any of the intra-operative exclusion criteria, and participant loss to follow-up (e.g. moves abroad/not contactable).

### Statistical methods

Results will be reported in accordance with the CONSORT guidelines for randomised controlled trials [21]. Analyses will be on an intention-to-treat (ITT) basis and will be conducted according to a pre-specified statistical analysis plan. The primary analyses will use data from all participants randomised who provided written informed consent, whose data is suitable (i.e. not missing). Any missing data will be reported, and data will be summarised as observed without imputation. Missing data values will be omitted from the primary analyses.

Continuous demographic variables such as age and BMI will be summated by the mean ± standard deviation if found to be normally distributed, and median with range or inter-quartile range otherwise. Categorical variables will be summarised by the number and percentage in each category.

To allow for differences in outcomes between participating surgeons, all analysis will be performed using generalised mixed (multilevel) models. Study group will be included as a fixed effect, with surgeon as a random effect. The primary outcome, the occurrence of trocar-site hernia over 2 years, will be analysed using a model assuming a binomial distribution with an identity link function. This will enable the percentage difference in outcome between groups to be calculated, which will be presented with a corresponding confidence interval. Secondary outcomes measured on a binary scale will be analysed using an equivalent approach to the primary outcome. Length of stay in hospital will be analysed using a linear mixed model. This outcome is expected to follow a positively skewed distribution and will thus be given a log transformation before analysis. All tests will be two-sided and a *p* value < 0.05 will be considered as statistically significant. Confidence intervals will be calculated at the 95% level.

A sensitivity analysis will be performed for the primary outcome. This will use multiple imputation for patients with missing outcome data. This will be performed using chained equations.

### Subgroup/exploratory analysis

We aim to conduct exploratory analysis of the primary outcome to investigate treatment effects in different patient subgroups. Subgroups will be BMI ≥ 30 versus < 30, diabetes versus no diabetes and emergency/expedited versus elective. The different effects of the intervention in patient subgroups will be explored by including subgroup and the subgroup by study group interaction in the mixed model. Where a significant interaction is found, the effects of the intervention in each subgroup will be quantified. Furthermore, patients in the control group will be analysed according to those who were closed with J-vicryl versus J-PDS.

### Data management

A Trial Steering Committee (TSC) has been established to oversee study conduct. The TSC comprises an independent chairperson (surgeon) and 3 other independent members (surgeon, statistician, and patient representative). The TSC have established formal terms of reference detailing its responsibilities and operational procedures.

An independent Data Monitoring Committee (DMC) has been established to review safety data, provide guidance on interim analyses and is advisory to the TSC. The DMC consists of a chairperson (statistician) and 3 other members (2 academic surgeons and 1 statistician) who are unblinded to participant treatment groups. Their duties and operational framework are described by terms of reference, defined from the DMC charter [22]. All members of the TSC, DMC, and the trial statistician are independent from the sponsor.

Both the TSC and DMC will hold a joint meeting before recruitment begins and will continue to convene every 6 months after recruitment has commenced. The DMC has the power to recommend continuation or termination of the study based on the evaluation of unblinded results.

### Integrity of data collection

Designated members of the research team will be responsible for data entry at different steps of the patient pathway. All researchers are trained to ensure the accuracy of the relevant outcome and will ensure that entries can be verified by the source data. Data will be collected pseudonymously and managed using Research Electronic Data Capture (REDCap) hosted by University College London (UCL). REDCap is a secure web-based software platform designed to support data capture for research studies on bespoke electronic case report forms (eCRFs). Unblinded research staff will deal with randomisation and per protocol operating, whereas blinded research staff will collect baseline information and primary and secondary outcome data.

### Audit procedures

The sponsor has confirmed Capacity and Capability to undertake the trial in line with Health Research Authority (HRA) guidance. This included an audit of site file documents including Good Clinical Practice (GCP) certificates and training records [23]. The sponsor will notify the Principal Investigator in advance if further internal audits are planned, typically at the halfway point and at study close.

### Protocol amendments

Substantial amendments require reporting to the REC in line with Health Research Authority guidance. Non-substantial amendments will be deliberated by the TSC and if deemed acceptable, notification will be provided to the Sponsor.

### Adverse event reporting

In this trial, adverse events (AE) are defined as any untoward medical occurrence in a subject to whom an intervention has been administered, including occurrences which are not necessarily caused by or related to the intervention and that exceed grade 1 of the Clavien-Dindo classification [24]. Serious adverse events (SAE) are defined as events which result in death, are life-threatening, require or prolong hospitalisation, or result in significant disability/incapacity. However, the following exceptions are already defined in this protocol and so will not be recorded as an adverse event; these are trocar-site hernia which will be assessed as a primary outcome, and wound events defined under SSO will be assessed as a secondary outcome.

We do not anticipate any device-specific AEs or SAEs. Suture granuloma and chronic draining sinuses are theoretically more common with non-absorbable suture material, but the largest published series of Duramesh implantation suggests these are rare, with an incidence of 0% and 0.3%, respectively [25]. Both will be reported as secondary outcomes, as stated above. AEs and SAEs will be reported unsolicited, and their relationship to the treatment arm graded as either unrelated, possible, probable or causal. Patients admitted to the hospital after their operation are reviewed daily by blinded outcome assessors for AEs, SAEs and wound events. Following discharge, trial participants are asked to inform the trial coordinator if they re-present to the hospital, whether that be related or unrelated to the index operation. Participants are also asked to contact the trial coordinator if they have any health-related concern so that AEs or SAEs are not missed.

### Study schema and interim analysis

**Fig. 1:**
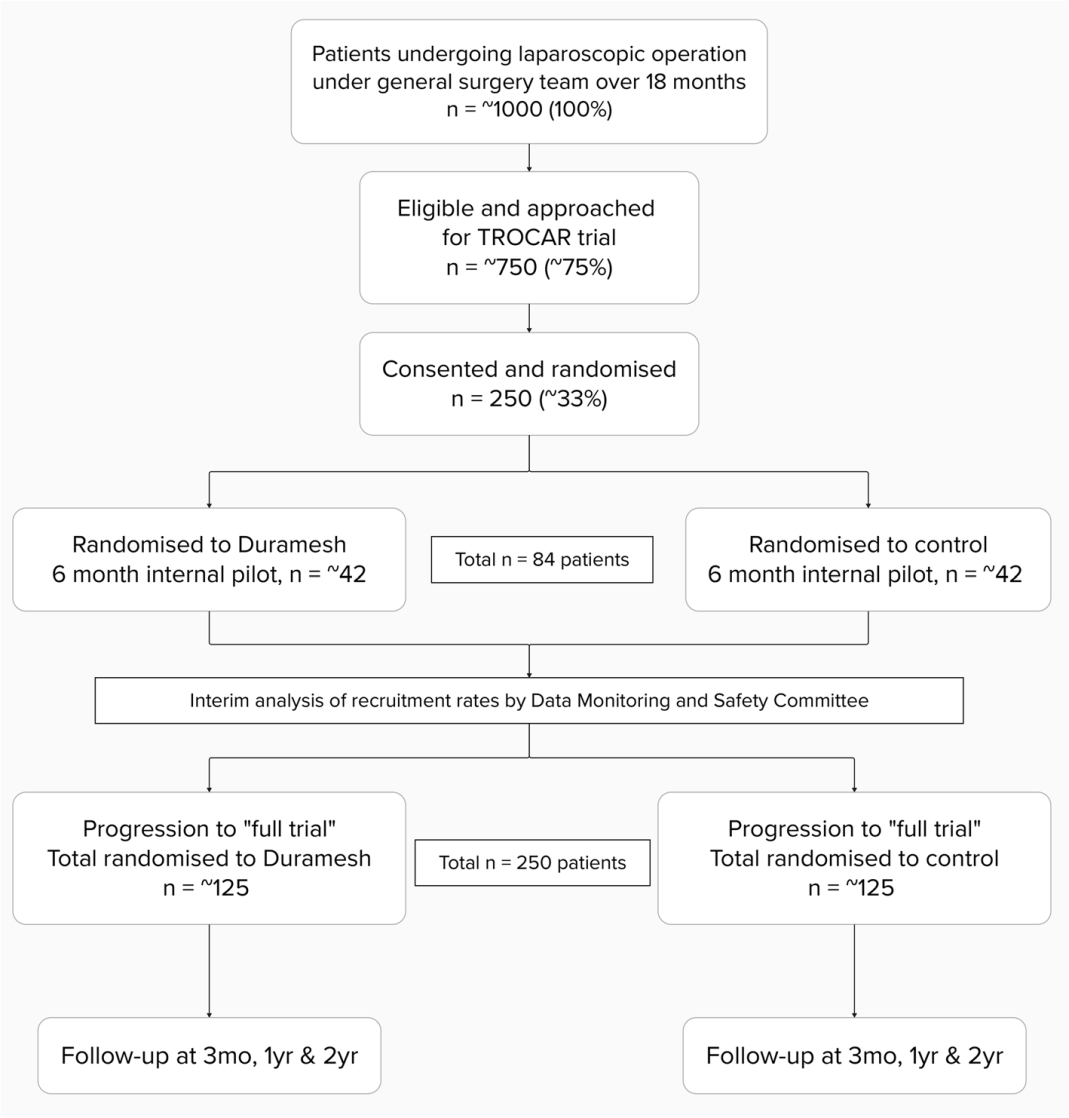
Study designed with an internal pilot with progression to the ‘full trial’ dependent on achieving adequate recruitment in 6 months

TROCAR has been designed with an internal pilot phase of 6 months’ duration (Fig. 1). During this time, we aim for 84 patients to be randomised. An interim analysis will be presented to the DMC after the internal pilot phase to confirm adequate recruitment and to monitor safety. A report will be compiled by the trial statistician, which will be presented to the DMC. The trial will continue to progress while the interim analysis is prepared. Participants from the internal pilot will contribute to the final analysis. In total, it is anticipated recruitment will take 12–18 months.

### Progression criteria

The internal pilot analysis will present total participants recruited, monthly recruitment rates and available secondary outcome data. Criteria for trial progression are outlined in Table 2. If all criteria are green, the trial will continue without protocol modification; if one or more criteria are amber, adaptations may be proposed to address the shortfall; one or more red criteria will require discussion about whether ongoing participant recruitment is feasible.

**Table 2:** Progression criteria stipulated at the interim analysis at 6 months

Criterion	Target	Green	Amber	Red
Participant recruitment	84	≥ 60	35–59	< 34
Randomisation rate (minimum number recruited per month)	14	≥ 9	5–8	< 5
Secondary outcome data available	80%	≥ 70%	50–70%	< 50%

In addition to recruitment analysis, the DMC will examine the unblinded frequency and severity of adverse events. If serious adverse events occur during the internal pilot that are assessed to be directly related to Duramesh and/or are disproportionately associated with Duramesh compared to conventional suture, then the DMC has the option to recommend trial termination. No specific number is declared, but judgement will be based on the severity of harm and its frequency relative to conventional suture.

### Indemnity arrangements

If there is negligent harm during the clinical trial when the NHS body owes a duty of care to the person harmed, NHS Indemnity covers NHS staff conducting the trial. NHS Indemnity does not offer no-fault compensation and is unable to agree in advance to pay compensation for non-negligent harm. The sponsor will provide post-trial care for complications or adverse events arising in trial patients following their operation.

### Data protection

Participant information will be kept confidential and managed in compliance with the Data Protection Act 2018, General Data Protection Regulations (GDPR), NHS Caldicott Principles, and the conditions of REC Approval. The Principal Investigator is responsible for maintaining strict confidentiality of locally stored trial documents, such as signed consent forms and quality of life questionnaires. The sponsor may require access to patient records for quality assurance and source data verification. In the event of specific issues or inquiries from regulatory authorities, access to the complete trial records may be necessary, provided patient confidentiality is safeguarded.

### Data storage

Following study end, patient-identifiable information will be kept for 3 years, after which all identifiable information, except for a list of local hospital numbers and corresponding unique study identifiers, will be confidentially destroyed. This list will be preserved as a method of record linkage to the research dataset to enable secondary research use in the future. Hard copies of study documentation such as quality-of-life questionnaires will also be destroyed by confidential means after transcribing onto REDCap. The full REDCap dataset will be moved to an encrypted version and archived indefinitely on UCL’s data safe haven.

### Publication policy

Research results will be published in peer-reviewed journals with the highest possible impact factor and those deemed appropriate by the PI. All proposed publications will be discussed with and reviewed by the TSC before publishing. Safety data will be reported descriptively and presented in the supplementary material.

### Data sharing

Access to an anonymised version of the final dataset will be made publicly available in the supplementary material. The full protocol and participant-level dataset will only be accessible for secondary research on reasonable request from the Principal Investigator after publication of the main study results. This is contingent upon the secondary research protocol obtaining a favourable opinion by a UK Research Ethics Committee or comparable ethics review body.

### Trial status

Ethical approval was received on 10th April 2025 by Cambridge Central Research Ethics Committee, reference number 25/EE/0057. The study was registered in the public domain and given International Standard Randomised Controlled Trial Number (ISRCTN) 14473961. The first TSC and DMC meeting was held in May 2025 with study termination rules and minor amendments to the protocol authorised. The latest version of the protocol is version 2.2. Recruitment began in June 2025 and is anticipated to complete by December 2026.

## Discussion

Trocar-site hernia (TSH) is a clinically important complication after laparoscopic surgery, with potential sequelae of pain and bowel incarceration. The TROCAR trial evaluates the comparative effectiveness of Duramesh versus conventional suture (J-Vicryl or J-PDS) for reducing the incidence of trocar-site hernia at periumbilical trocar sites. The hypothesis is that Duramesh is superior to conventional suture.

The mechanism of action of Duramesh is by distributing tension at the suture–tissue interface and by promoting fibrovascular integration between its polypropylene filaments [11]. This theoretically reduces fascial tearing and subsequent hernia formation. Preclinical studies in animal models have shown greater resistance to suture pull-through and greater ultimate tensile strength before tearing [12, 26]. Our team performed a systematic review of clinical studies [13] which suggested Duramesh may be associated with low rates of incisional hernia, but the available evidence remains limited by study quality and high risk of bias. Randomised controlled trials (RCTs) are therefore needed before widespread adoption can be recommended.

The INDURATE trial, currently recruiting in Madrid [27], also investigates Duramesh closure for periumbilical trocar-sites. However, its design includes certain constraints, including the absence of blinded outcome assessment, relatively short follow-up duration (1 year), and the assumption of large absolute risk reduction for power calculation (20%). This results in a small sample size of 124 participants after accounting for a 5% rate of loss to follow up. Other RCTs are also currently recruiting, but these investigate Duramesh closure of laparotomy and ileostomy wounds [28, 29] and are not designed with patient or outcome assessor blinding. TROCAR therefore complements ongoing research and is positioned to be the first double-blind and adequately powered trial for TSH.

However, results of this trial may not be applicable to certain patient subgroups such as those with severe obesity (BMI > 40) or colorectal cancer. BMI ≥ 40 was specifically chosen as an exclusion criterion because Duramesh is only available with a ½ circle needle. Without a J-shaped needle, implantation can be technically challenging due to greater depth of subcutaneous fat. In addition, larger incisions are more prone to hernia occurrence which undermines direct comparison of trocar incisions with mini-laparotomy incisions for specimen extraction. Whilst colectomy specimens can be extracted through a separate Pfannenstiel incision, at our institution, extending the periumbilical trocar site (> 3 cm) is the favoured approach. Hence, most colectomy and anterior resection operations will be excluded.

TSH is likely an underappreciated complication of laparoscopic surgery. This is because most systematic reviews are derived from retrospective studies that only report TSH as a secondary outcome based on clinical examination or reoperation. Studies dedicated to TSH assessment as the primary outcome found a pooled incidence of 24.5% [30]. Imaging for detecting TSH as the primary outcome is more sensitive than physical examination, as subclinical hernias are easily missed in obese patients. In this study, ultrasound was chosen over CT scan due to centre-specific logistical considerations and concerns about ionising radiation. Since ultrasound is operator dependent, findings may be susceptible to bias. To mitigate this, a standard procedure will be followed, double blinding as previously described, and images of equivocal findings captured for independent adjudication. Moreover, TROCAR is an investigator-initiated trial with all outcomes and AE/SAEs reported by researchers independent of the funding body.

Whilst hernia occurrence is an important surgical outcome, we acknowledge the clinical relevance of an occult hernia (radiologically detectable) is debatable and have therefore chosen to investigate quality of life (QoL) as well. However, there is a paucity of QoL measures in the literature specific to hernia occurrence after primary surgery. We therefore use modified versions of the EuraHS-QoL score [16] and Carolinas Comfort Scale (CCS) [17, 18] which are validated for ventral hernia repair. These represent the best available tools for assessment of hernia-related symptoms. EuraHS-QoL is free to use, whereas CCS holds a trademark and requires a licensing agreement (obtained for this trial). We made only minor changes to the wording so that patient-reported outcomes are specific to the umbilical region rather than hernia repair site.

In summary, TROCAR will provide level 1 evidence investigating whether the use of Duramesh prevents the formation of periumbilical trocar-site incisional hernia after laparoscopic surgery in both the emergency and elective settings.

## Supplementary Information

Supplementary Material 1

## Data Availability

The sponsor maintains overall responsibility for the final dataset. An anonymised version will become available in the supplementary materials in line with open science expectations.
